# Supplementary material for: Optimal Design of Galvanic Vestibular Stimulation for Patients with Vestibulopathy and Cerebellar Disorders
Source: Brain Sci. 2023 Sep 16;13(9):1333. doi: 10.3390/brainsci13091333 (PMC10526825; doi:10.3390/brainsci13091333)
Supplement: Supplementary file 1 [file brainsci-13-01333-s001.zip › brainsci-2551645-supplementary.pdf]

Supplementary Table S1. Representation of the appearance of spontaneous spinning or tilting sensation in patients when applying GVS

Pt.	Classification	Modes																	
		**Sinusoidal**						**Noisy**						**Direct current (DC)**					
		**1**	**2**	**3**	**4**	**5**	**6**	**7**	**8**	**9**	**10**	**11**	**12**	**13**	**14**	**15**	**16**	**17**	**18**
1	Acute UVP	-	-	-	-	-	-	-	-	-	-	-	-	-	-	-	-	+	+
2	Acute UVP	-	-	-	-	-	-	-	-	-	-	-	-	-	-	-	+	-	+
3	Acute UVP	-	-	-	-	-	-	-	-	-	-	-	-	-	-	-	-	-	-
4	Acute UVP	-	-	-	-	-	-	-	-	-	-	-	-	-	-	-	-	+	+
5	Acute UVP	-	-	-	-	-	-	-	-	-	-	-	-	-	-	-	-	+	-
6	Acute UVP	-	-	-	-	-	-	-	-	-	-	-	-	-	-	-	-	-	-
7	Acute UVP	-	-	-	-	-	-	-	-	-	-	-	-	-	-	-	-	-	+
8	Chronic UVP	-	-	-	-	-	-	-	-	-	-	-	-	-	-	-	-	-	+
9	Chronic UVP	-	-	-	-	-	-	-	-	-	-	-	-	-	-	-	-	-	-
10	Chronic BVP	-	-	-	-	-	-	-	-	-	-	-	-						
11	Chronic BVP	-	-	-	-	-	-	-	-	-	-	-	-						
12	Chronic BVP	-	-	-	-	-	-	-	-	-	-	-	-						
13	Chronic BVP	-	-	-	-	-	-	-	-	-	-	-	-						
14	Chronic BVP	-	-	-	-	-	-	-	-	-	-	-	-						
15	Chronic BVP	-	-	-	-	-	-	-	-	-	-	-	-						
16	Chronic BVP	-	-	-	-	-	-	-	-	-	-	-	-						
17	Chronic BVP	-	-	-	-	-	-	-	-	-	-	-	-						
18	Chronic BVP	-	-	-	-	-	-	-	-	-	-	-	-						
19	CA	-	-	-	-	-	-	-	-	-	-	-	-						
20	CA	-	-	-	-	-	-	-	-	-	-	-	-						
21	CA	-	-	-	-	-	-	-	-	-	-	-	-						
22	CA	-	-	-	-	-	-	-	-	-	-	-	-						
23	CA	-	-	-	-	-	-	-	-	-	-	-	-						
24	CA	-	-	-	-	-	-	-	-	-	-	-	-						
25	CA	-	-	-	-	-	-	-	-	-	-	-	-						
26	CA	-	-	-	-	-	-	-	-	-	-	-	-						
27	CA	-	-	-	-	-	-	-	-	-	-	-	-						
28	CA	-	-	-	-	-	-	-	-	-	-	-	-						
29	CA	-	-	-	-	-	-	-	-	-	-	-	-						
30	CA	-	-	-	-	-	-	-	-	-	-	-	-						
31	CA	-	-	-	-	-	-	-	-	-	-	-	-						

UVP=unilateral vestibulopathy; BVP=bilateral vestibulopathy; CA=cerebellar ataxia; + indicates the presence of a spinning or tingling sensation; - indicates the absence of a spinning or tingling sensation
